# 53 The development of a multi-institutional prospective registry for patients with metastatic invasive lobular carcinoma: identifying new markers of disease progression – CORRIGENDUM

**DOI:** 10.1017/cts.2023.637

**Published:** 2023-09-28

**Authors:** Harriet T Rothschild, A Jo Chien, Rachel C Jankowitz, Mark JM Magbanua, Jason A Mouabbi, Rebecca A Shatsky, Julia Levine, Rita A Mukhtar

The above abstract published with mistakes in some author names and affiliations. Some authors names were not included in the author list at all.

The correct list of authors and their affiliations is as follows:

Harriet T Rothschild^1^, A Jo Chien^2^, Rachel C Jankowitz^3^, Mark JM Magbanua^4^, Jason A Mouabbi^5^, Rebecca A Shatsky^6^, Julia Levine^7^ and Rita A Mukhtar^8^

^1^School of Medicine, University of California, San Francisco, ^2^Department of Medicine, University of California, San Francisco, ^3^Department of Medicine, University of Pennsylvania, ^4^Department of Laboratory Medicine, University of California, San Francisco, ^5^Department of Breast Medical Oncology, MD Anderson Cancer Center, ^6^Department of Medicine, University of California, San Diego, ^7^Lobular Breast Cancer Alliance and ^8^Department of Surgery, University of California, San Francisco

The original abstract has been corrected online to rectify these errors.
